# Influence of Y_2_O_3_ Nano-Dispersoids on the Characteristics of AlCoCrFeNi_2.1_-Reinforced Tungsten Alloys via Mechanical Alloying and Low-Temperature Sintering

**DOI:** 10.3390/ma18030672

**Published:** 2025-02-03

**Authors:** Chun-Liang Chen, Fang-Yu Huang, Geoff West

**Affiliations:** 1 Department of Materials Science and Engineering, National Dong Hwa University, Hualien 97401, Taiwan; 2 Warwick Manufacturing Group (WMG), University of Warwick, Coventry CV4 7AL, UK

**Keywords:** tungsten composites, high-entropy alloys, dual-phase structure, mechanical alloying, secondary phase

## Abstract

This study investigates the effects of nano-oxide dispersoids on microstructural evolution, phase formation, and mechanical properties of W-Mo-Ti alloys reinforced with AlCoCrFeNi_2.1_ during mechanical alloying. An EBSD/EDS analysis confirmed the formation of different phases, including the tungsten matrix, FCC reinforcement phase, Al_2_O_3_, and (Al,Cr) oxide. Y_2_O_3_ particles played a crucial role in refining the microstructure, promoting a uniform dispersion of the reinforcement phase and oxide particles in the tungsten model alloys. Mechanical testing demonstrates that the Y_2_O_3_-containing alloy exhibits improved hardness with prolonged milling, attributed to the refinement in the microstructure. In contrast, the Y_2_O_3_-free alloy shows reduced hardness due to the agglomeration of reinforcement phases surrounded by an (Al,Cr) oxide layer. The model tungsten alloys exhibit brittle behavior in compression tests, which can be attributed to the presence of (Al,Cr) oxide layers weakening the interfacial bonding and limiting plastic deformation.

## 1. Introduction

Tungsten-based alloys exhibit outstanding high-temperature stability, excellent thermal conductivity, and good corrosion resistance and, therefore, are ideal structural materials for applications in the aerospace, nuclear, and medical industries [1,2]. Small amounts of Mo and Ti are commonly added to tungsten-based alloys to enhance solid solution strengthening. However, their inherent brittleness limits their applications, such as a high ductile-to-brittle transition temperature (DBTT) and recrystallization-induced brittleness [3]. Traditionally, low-melting-point elements (e.g., Ni, Fe, and Co) are commonly used as binder phases to improve the ductility of tungsten-based alloys. In recent years, several studies have reported the use of high-entropy alloys (HEAs) in binder or reinforcement phases in tungsten composites [4,5,6]. The addition of HEAs significantly enhances the mechanical properties of tungsten-based alloys, a contribution largely attributed to the four core effects of HEAs: the high-entropy effect, lattice distortion effect, cocktail effect, and sluggish diffusion effect [7]. Eutectic high-entropy alloys (EHEAs) are characterized by their lamellar microstructure and are highly valued for their excellent castability, phase stability, and mechanical properties [8,9,10]. In the design of EHEAs, similar eutectic reactions have been observed across various dual-phase systems, which in some cases can even form pseudo-binary systems [11,12]. Among these materials, AlCoCrFeNi_2.1_ stands out as one of the most promising EHEAs that has attracted significant research attention [13,14,15]. Its microstructure generally consists of a disordered FCC phase enriched with Co, Cr, and Fe and an ordered BCC B2 phase dominated by Al and Ni [16,17,18]. The dual-phase structure, consisting of BCC and FCC, offers a good combination of strength and ductility to alloys [13,19].

Additionally, oxide dispersion strengthening (ODS) is a critical mechanism for high-temperature materials, relying on highly stable oxide particles, such as Y_2_O_3_, that remain resistant to decomposition during processing at elevated temperatures [20,21,22,23,24]. Mechanical alloying (MA) is a solid-state processing technique that involves high-energy ball milling, which facilitates repeated cold welding and fracturing of materials at relatively low temperatures. This method enables the refinement of the microstructure and the uniform dispersion of high-temperature-stable oxide particles in materials [25]. Therefore, MA is an ideal method for producing tungsten-based alloys with the addition of nano-oxide dispersed particles.

In earlier studies, tungsten-based alloys reinforced with AlCoCrFeNi and CoCrFeNiTa EHEAs were investigated [26], demonstrating that the stability of the reinforcement phases strongly depends on the sintering temperature due to phase decomposition occurring during high-temperature sintering. Furthermore, the fine dispersion of Y_2_O_3_ particles influences microstructural evolution by pinning grain boundaries and refining the overall grain structure. They also enhance material stability by inhibiting phase decomposition during high-temperature sintering, acting as effective thermal stabilizers. Therefore, it is crucial to further investigate the effects of sintering temperatures and how oxide dispersoids influence and stabilize the formation of reinforcement phases in tungsten-based alloys, improving their mechanical properties. This study begins with an investigation of AlCoCrFeNi_2.1_ alloys, followed by the examination of tungsten alloys reinforced with AlCoCrFeNi_2.1_ and dispersed with Y_2_O_3_. The tungsten model alloys were fabricated using mechanical alloying followed by low-temperature sintering. The objective of this research is to investigate the role of Y_2_O_3_ oxide particles in phase formation, microstructure evolution, and the mechanical properties of tungsten-based alloys, with a particular focus on the effect of Y_2_O_3_ on phase stability and its interaction between the tungsten alloy matrix and the reinforcement phase.

## 2. Materials and Methods

In this work, raw powders of W, Mo, Ti, Al, Co, Cr, Fe, and Ni with a purity greater than 99.9% and particle sizes of approximately 45 μm were used. The Y_2_O_3_ dispersoids had a nanoscale size ranging from 20 to 50 nm and a purity of 99.99%. All powders were commercially sourced and supplied by Ultimate Materials Technology Co., Ltd., Taiwan. Firstly, the AlCoCrFeNi_2.1_ high-entropy alloys, selected as reinforcement phases, were fabricated and analyzed. The matrix phase, composed of tungsten powders doped with Mo, Ti, and Y_2_O_3_, was pre-milled for 8 h, while the reinforcement phase, consisting of AlCoCrFeNi_2.1_ powders, underwent pre-milling for 16 h. The tungsten-based matrix powders were subsequently ball-milled with AlCoCrFeNi_2.1_ powders for durations of 4, 8, and 16 h to synthesize the tungsten-based composite powders. Fifteen weight percent of AlCoCrFeNi_2.1_ was introduced into the tungsten-based matrix. Two tungsten model alloys were designed: 81W-3Mo-1Ti-15(AlCoCrFeNi_2.1_), referred to as “W-Al”, and 79W-3Mo-1Ti-2Y_2_O_3_-15(AlCoCrFeNi_2.1_), referred to as “W-Al-Y.” The detailed compositions of these alloys are presented in Table 1. Powder mixing was conducted in an argon-filled glovebox to prevent oxidation. Mechanical alloying was carried out using a Retsch PM100 planetary mill operating at 300 rpm with a ball to powder weight ratio (BPR) of 20:1. The milled powders were subsequently pressed into a die under a pressure of 210 MPa. Finally, the green compacts were sintered at 1250 °C for one hour under a high vacuum of 10^−5^ torr. Phase formation and microstructural changes in both powders and bulk materials at various stages were characterized using XRD, SEM, and EBSD, with an EDS suite for chemical composition analysis of the consolidated samples. EBSD and EDS mapping data were acquired using a Versa 3D FEG-SEM equipped with an Oxford Instruments Symmetry 2 EBSD detector and an Ultim Max 170 EDS detector. The measurements were conducted at 20 kV with a step size of 0.1 μm and a collection speed of 250 Hz. Data acquisition and processing were performed using Aztec 5.0 and Crystal 2.0 software. The samples were prepared for EBSD analysis using standard metallographic techniques. Mechanical polishing was carried out with diamond suspensions (6 μm and 1 μm), followed by a final polishing step with a 0.05 μm colloidal silica suspension. To minimize surface damage and enhance the quality of EBSD data, the preparation was completed with ion beam milling using argon ions. Room temperature compressive tests were conducted at a strain rate of 10^−3^ s^−1^ using a 100 kN universal testing machine (SHIMADZU, AGS-100kNX, Kyoto, Japan), following the guidelines of ASTM E9. The compression sample dimensions were a diameter of 6 mm and a height of 8 mm. Vickers hardness measurements (FUTURE-TECH CORP, FM-310e, Kawasaki, Japan) were performed using a 0.025 kgf load applied for 15 s, corresponding to HV0.025, in accordance with the ASTM E384 standard.

## 3. Results and Discussion

### 3.1. Characterization of the AlCoCrFeNi_2.1_ Reinforcement

#### 3.1.1. SEM

**Figure 1:**
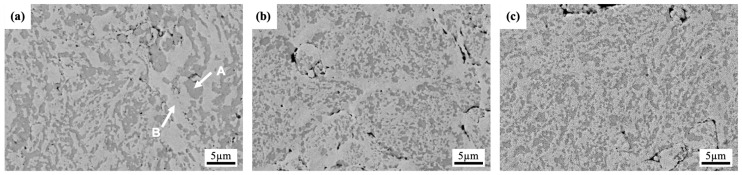
SEM images of AlCoCrFeNi_2.1_ alloys milled for (**a**) 4 h, (**b**) 8 h, and (**c**) 16 h.

Figure 1 shows the microstructural evolution of the AlCoCrFeNi_2.1_ alloy with increasing milling time. After 4 h of milling, the microstructure exhibits a relatively coarse-grained structure with a heterogeneous distribution of phases, as shown in Figure 1a. The regions with gray contrast exhibit high levels of Al and Ni, corresponding to the formation of the ordered AlNi-BCC phase (see point “A”). On the contrary, the bright areas in the SEM image represent the disordered FCC phase, mainly composed of Co, Cr, Fe, and Ni (refer to point “B”). The EDS analysis results for the different phases, identified by various labeled points in the model alloys, are presented in Table 2. The AlNi-BCC phase is embedded within the FCC matrix and appears to be more clustered in the early stage of milling. The black areas observed in the microstructure are voids or pores, which contain Al-rich oxides that formed as a result of the oxidation of aluminum during the milling process.

The microstructure becomes more refined after 8 h of milling, suggesting that higher milling intensity was greatened and accelerated the process of grain refinement; see Figure 1b. The AlNi BCC phase still tends to cluster or agglomerate on the FCC matrix, which may indicate incomplete dispersion or limited solubility between the phases at this stage of milling. At the final stage of milling, see Figure 1c, the microstructure shows further refinement, and the AlNi BCC phase is more uniformly dispersed throughout the FCC matrix. The repeated cycles of cold welding and fracturing during prolonged milling contribute to the refinement of the microstructure and the homogenization of the phase distribution.

#### 3.1.2. EBSD/EDS Mapping

**Figure 2:**
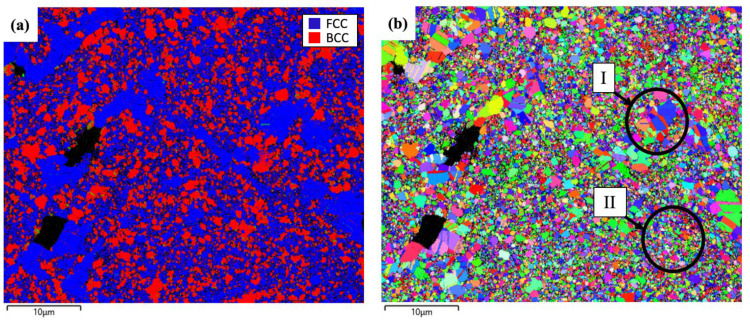
EBSD investigations of the AlCoCrFeNi_2.1_ alloy: (**a**,**b**) EBSD phase map and EBSD orientation map.

Figure 2 shows the results of an EBSD analysis of the AlCoCrFeNi_2.1_ alloy after 16 h of milling, providing critical information for the phase distribution and crystallographic orientation of the alloy. The EBSD phase map, see Figure 2a, illustrates the distribution of different phases in the alloy. The dominant phase is the disordered FCC structure, highlighted in blue, consisting of Cr, Fe, and Co, which serves as the matrix phase. In addition to the FCC phase, rounded particles dispersed throughout the matrix represent BCC phases (red color). These particles are identified as ordered AlNi BCC (B2) or disordered Cr-rich BCC (A2) phases. Due to the similar crystal structures of these phases, EBSD alone is insufficient to differentiate the phases. Further phase identification of the BCC phases can be confirmed using EDS mapping analysis based on elemental composition. The BCC phases play a role in reinforcing the FCC matrix and influencing the hardness and strength of the alloys.

Figure 2b displays the EBSD orientation map of the alloy. It reveals that the alloy demonstrates a variety of grain sizes, morphologies, and orientations. The heterogeneity of the alloy is a characteristic feature of dual-phase structured materials. It is evident that the FCC phase contains grains with twinned structures, particularly within the larger grains (see position “I” in Figure 2b). The observed twinning behavior suggests the presence of deformation that is characteristic of ductile FCC alloys with low stacking fault energy [27], contributing to a good balance between the strength and ductility of the alloys. Additionally, the smaller grains of the FCC matrix phase exhibit a nanostructure, with grain sizes approaching ~200 nm (see position “II” in Figure 2b). The fine-grained structure is a result of severe plastic deformation during mechanical alloying, which enhances the strength of the alloy.

**Figure 3:**
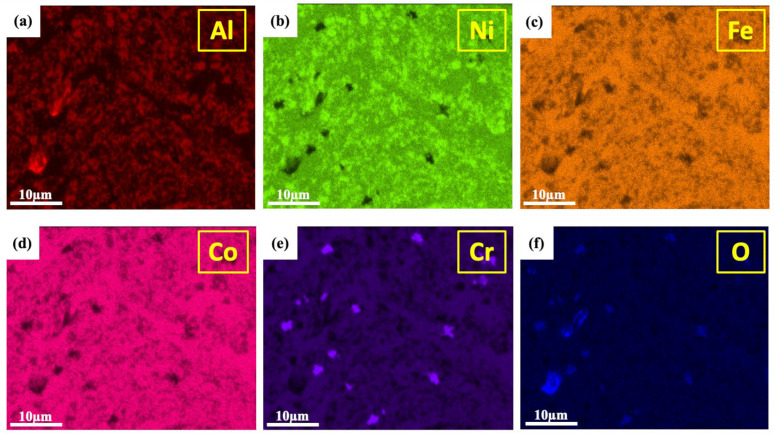
EBSD-EDS mapping of the AlCoCrFeNi_2.1_ alloy: (**a**) Al map, (**b**) Ni map, (**c**) Co map, (**d**) Co map, (**e**) Cr map, and (**f**) O map.

Figure 3 presents an EDS mapping analysis, which provides critical compositional information and distinguishes the phases in the AlCoCrFeNi_2.1_ alloy. The AlNi BCC phase is identified by its high Al and Ni concentrations (see Figure 3a,b), which correspond to the ordered BCC (B2) phase. Similarly, the disordered Cr-rich BCC (A2) phase is characterized by an elevated Cr content (see Figure 3e), distinguishing it from the AlNi BCC phases. Despite the similarity in their crystal structures, the compositional differences between these two BCC phases allow for their differentiation through EDS mapping. The FCC phase shows a clear enrichment of Fe and Co elements, as seen in the corresponding maps (see Figure 3c,d). Regions with a high concentration of Al and O also indicate the presence of Al-rich oxides. Al has a high affinity for oxygen, making it prone to forming oxides during the process. The formation of these oxides is likely due to oxygen contamination, which commonly occurs during mechanical alloying, particularly in fine-grained structures with high surface areas subjected to high-temperature sintering.

### 3.2. Characterization of the W-Al Model Alloy

#### 3.2.1. XRD Analysis of Milled Powders

**Figure 4:**
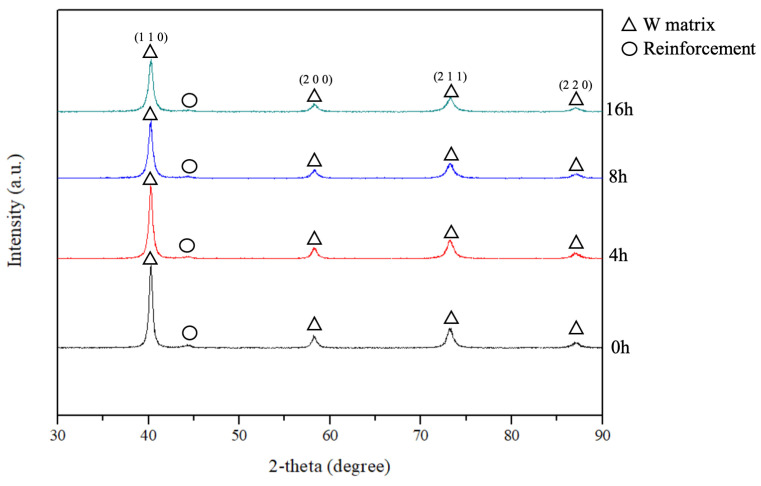
XRD patterns of the W-Al model alloy powders after different milling durations.

Figure 4 shows the XRD patterns of the W-Al alloy powders at different milling durations. The main peaks correspond to the tungsten matrix, associated with the (110), (200), (211), and (220) planes, and remain dominant throughout the milling process. The broadening of tungsten peaks and the reduction in their intensities with increased milling time are attributed to the introduction of crystal defects and lattice strain in tungsten, arising from collision events during ball milling. A weak peak at a 2-theta angle of approximately 43° is observed, which corresponds to the presence of the reinforcement phase (AlCoCrFeNi_2.1_). As the milling time increases, the reinforcement phase increasingly interacts with the tungsten matrix phase, leading to the gradual formation of a partial solid solution. The milling process further contributes to peak broadening as the atomic lattice becomes more disordered due to the dissolution of the reinforcement phase atoms into the tungsten matrix.

#### 3.2.2. SEM

**Figure 5:**
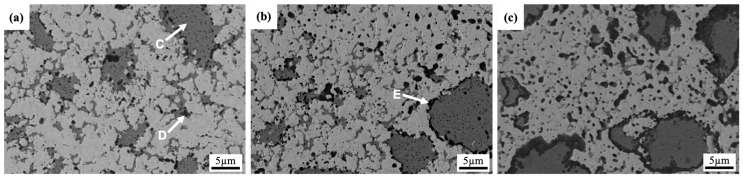
SEM images of W-Al model alloys milled for (**a**) 4 h, (**b**) 8 h, and (**c**) 16 h.

Figure 5 shows the SEM images of the tungsten alloys reinforced with AlCoCrFeNi_2.1_ after different milling times of 4 h, 8 h, and 16 h. The reinforcement phase of AlCoCrFeNi_2.1_ appears in a gray contrast (see point “C” in Figure 5a) and is dispersed in the tungsten matrix. In the initial stage of milling (4 h), the reinforcement phase exhibits a nonuniform distribution and significant size variation in the tungsten matrix. Smaller grains in the reinforcement phase are observed growing between the tungsten grains. It suggests that the reinforcement phase has been successfully embedded within the tungsten matrix during the early stages of the milling process. In addition, dark particles are identified as Al-rich oxides (see point “D” in Figure 5a) in the microstructure, suggesting that the milling process promotes the oxidation of aluminum in the AlCoCrFeNi_2.1_ phase. As the milling time increased to 8 h, the formation of (Al,Cr) oxides around the AlCoCrFeNi_2.1_ reinforcement phase became more pronounced (see point “E” in Figure 5b). This indicates that prolonged milling facilitates oxidation around the reinforcement phase due to the increased interaction with residual oxygen during the ball milling process. With further milling up to 16 h, the microstructure shows increased agglomeration and coarsening of the AlCoCrFeNi_2.1_ reinforcement phases. This suggests that extended milling causes the reinforcement particles to coalesce, resulting in larger clusters. Additionally, an (Al,Cr) oxide layer surrounds the reinforcement phases, which may be due to diffusion or further oxidation during the prolonged milling process. A thicker layer of (Al,Cr) oxides can thus form a shell around the reinforcement phases. It is believed that prolonged milling also causes repeated fracturing and cold welding of particles, which promote fresh aluminum surfaces to oxygen and facilitate further oxide layer formation around the reinforcement phases.

#### 3.2.3. EBSD/EDS Mapping

**Figure 6:**
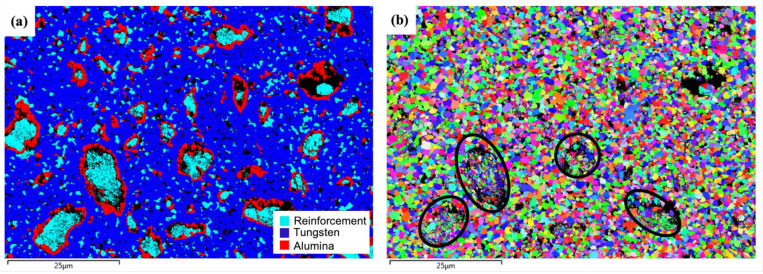
EBSD investigations of the W-Al model alloy: (**a**,**b**) EBSD phase map and EBSD orientation map.

Figure 6 presents the EBSD analysis of the W-Al model alloy. The EBSD phase map in Figure 6a reveals three distinct phases in the microstructure. The tungsten matrix is identified by blue regions. The AlCoCrFeNi_2.1_ reinforcement phases appear as light blue areas and are confirmed to have an FCC structure. Al-rich oxides are marked in red and tend to form around larger reinforcement particles. The tungsten matrix is the primary phase, constituting 63.7% of the volume fraction, followed by the AlCoCrFeNi_2.1_ reinforcement phase (12.5%) and alumina phase (9.63%). The reinforcement phases show a nonuniform distribution, with larger particles (5~15 um) surrounded by (Al,Cr) oxide layers and smaller particles (~1 um) dispersed more uniformly within the tungsten matrix. The different distribution phenomenon suggests that the formation and growth of the reinforcement phase are influenced by diffusion kinetics and interfacial reactions. In this case, aluminum depletion from the reinforcement phase instead leads to the formation of (Al,Cr) oxide layers around larger reinforcement particles. This depletion can hinder the formation of the AlNi BCC phase, which is typically observed in AlCoCrFeNi alloys.

The EBSD orientation map demonstrates a random orientation distribution of tungsten matrix grains, suggesting no preferential crystallographic alignment in the matrix, as shown in Figure 6b. The average grain size of the reinforcement phase (d_FCC_ = 0.50 µm ± 0.21) and the tungsten matrix (d_W_ = 1.0 µm ± 0.47) were measured, respectively. The large reinforcement phases with an FCC structure exhibit a fine-grained microstructure, as indicated by the black circles in Figure 6b, suggesting that extensive plastic deformation occurred during the milling process.

**Figure 7:**
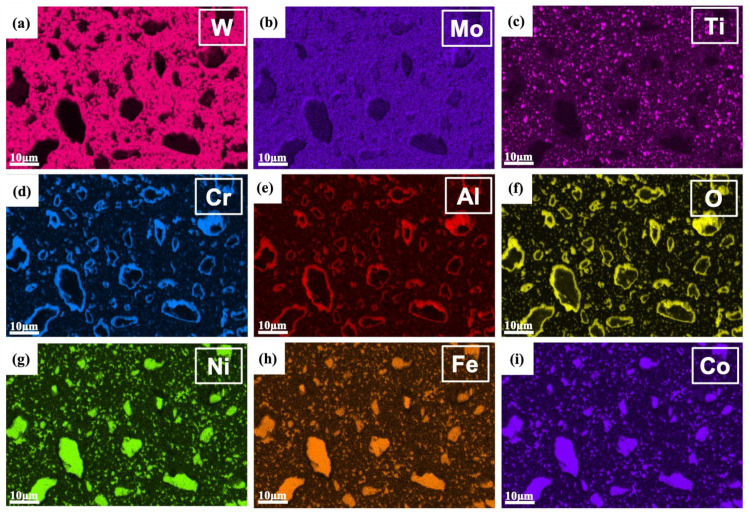
EBSD-EDS mapping of the W-Al model alloy: (**a**) W map, (**b**) Mo map, (**c**) Ti map, (**d**) Cr map, (**e**) Al map, (**f**) O map, (**g**) Ni map, (**h**) Fe map, and (**i**) Co map.

Figure 7 shows the EDS mapping of the W-Al model alloy, which provides the elemental distribution and phase identification of the sample alloy. The tungsten matrix phase is apparently identified by high levels of W and Mo elements in the corresponding EDS maps, see Figure 7a,b. It indicates that the matrix is primarily composed of tungsten, with Mo acting as a solid solution element and stabilizer. In addition, the regions with high concentrations of Ni, Fe, and Co are associated with the formation of the AlCoCrFeNi_2.1_ reinforcement phase, which could contribute to the strength and toughness of the tungsten composite. The small clustering of Ni, Fe, and Co in microstructure indicates a uniform dispersion of the fine reinforcement phase in the tungsten matrix. Conversely, areas with elevated levels of Al, Cr, and O are indicative of (Al,Cr) oxide formation, which is clearly observed surrounding the larger reinforcement particles. This finding aligns with the EBSD phase map, which reveals (Al,Cr) oxides surrounding the large reinforcement particles. The formation of these oxides results from the depletion of Al from the reinforcement phase, which then reacts with Cr and O to form oxide layers. These layers may serve as barriers surrounding the reinforcements, thereby affecting the mechanical properties. The presence of Ti-rich oxides is indicated by the distribution of Ti and O, suggesting that titanium also reacted with oxygen during the processing. These oxides are smaller in size (< 1 um) and do not preferentially segregate around reinforcement particles, but instead disperse evenly throughout the tungsten matrix. It should be noted that the uniform oxide dispersion may provide additional strengthening and stabilization effects due to the oxide dispersion strengthening (ODS) mechanism.

### 3.3. Characterization of the W-Al-Y Model Alloys

#### 3.3.1. SEM

**Figure 8:**
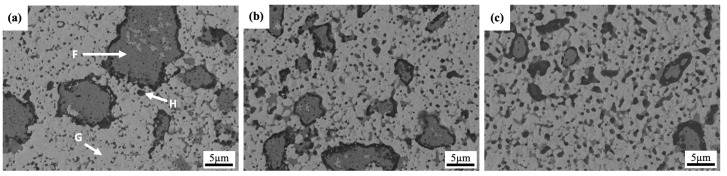
SEM images of W-Al-Y model alloys milled for (**a**) 4 h, (**b**) 8 h, and (**c**) 16 h.

Figure 8 illustrates the microstructural evolution of the W-Al-Y model alloys during mechanical alloying. In the initial stage of milling, the SEM image, Figure 8a, shows large AlCoCrFeNi_2.1_ reinforcement phases (see point “F”) embedded in a tungsten matrix (see point “H”), with (Al,Cr) oxides surrounding the reinforcement phases. The formation of these large reinforcement phases and the presence of Al-rich oxides (see point “G”) can be attributed to the influence of nano-Y_2_O_3_ particles during ball milling. In this case, nano-Y_2_O_3_ particles can act as nucleation sites for the formation and growth of reinforcement phases. During processing, these nano-oxides may attract alloying elements that tend to form reinforcements, causing the reinforcement phases to precipitate and grow around the Y_2_O_3_ particles. However, after only 4 h of milling, the mechanical alloying process may be insufficient for achieving a uniform distribution of nano-Y_2_O_3_ particles. This incomplete ball milling can cause the reinforcement elements to concentrate around specific regions, leading to the early formation of larger reinforcement phases instead of a fine and uniform distribution throughout the tungsten matrix.

In addition, at the beginning of milling, nano-Y_2_O_3_ particles, due to their high surface energy, may not disperse uniformly, and instead tend to agglomerate. These agglomerated Y_2_O_3_ particles can attract other alloying elements, such as Al, Ni, and Fe, from the AlCoCrFeNi_2.1_ phase. This clustering effect encourages the formation of larger reinforcement particles around the Y_2_O_3_ agglomerates. On the other hand, the (Al,Cr) oxide layer was observed surrounding the large reinforcement phases at the early stage of milling, a phenomenon not seen in the Y_2_O_3_-free sample (see Figure 5a). In this case, Y_2_O_3_ particles can also act as nucleation sites to promote the formation of a stable Al-Y-O phase, which is thermodynamically favorable due to the strong bonding between aluminum, yttrium, and oxygen. After prolonged milling times (8 h and 16 h), the microstructure becomes finer-grained, with a significant reduction in the size of the reinforcement phases. Additionally, a distinct (Al,Cr) oxide layer surrounds the large reinforcement phases, and it appears to thicken with extended milling times. This thickening is attributed to the high-impact energy at the surface of the reinforcement phases, which accelerates the formation of oxidation. Furthermore, the small dark particles, which could be either Al-rich or Ti-rich oxides, increase in number after long milling durations. The small reinforcement phases and dark oxide particles are uniformly dispersed throughout the tungsten matrix. The results suggest that the repeated collisions during milling facilitate uniform dispersion of the particles, minimizing the clustering or agglomeration of the reinforcement phases that have been present initially.

**Table 2:** EDS analysis of the AlCoCrFeNi_2.1_ and tungsten model alloys (at.%).

Material	Position	O	Ti	Al	Cr	Fe	Co	Ni	Mo	W	Phase
AlCoCrFeNi_2.1_ (Figure 1)	A	-	-	31.31	4.44	8.26	11.96	44.03	-	-	BCC
	B	-	-	5.35	20.21	20.10	21.02	33.33	-	-	FCC
W-Al (Figure 5)	C	-	-	-	-	22.91	25.66	50.03	-	1.40	Reinforcement
	D	65.56	1.19	12.05	15.59	-	-	1.20	-	4.41	Al-rich oxide
	E	52.65	-	18.58	3.69	4.79	5.10	12.50	-	2.68	Oxide layer
W-Al-Y (Figure 8)	F	-	-	-	-	17.14	17.83	54.62	-	10.41	Reinforcement
	G	-	-	-	-	-	-	-	7.97	92.03	W matrix
	H	68.78	1.96	24.10	-	-	-	1.01	-	4.14	Al-rich oxide

#### 3.3.2. EBSD/EDS Mapping

**Figure 9:**
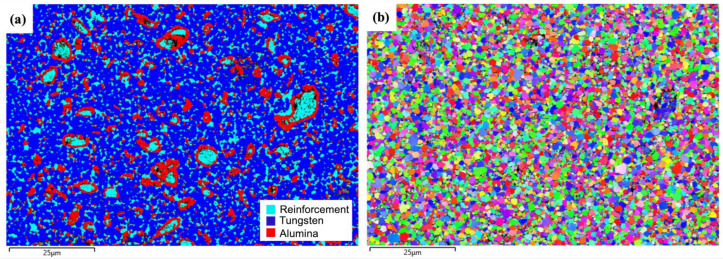
EBSD investigations of the W-Al-Y model alloy: (**a**,**b**) EBSD phase map and EBSD orientation map.

EBSD analysis of the W-Al-Y model alloy is presented in Figure 9. In the EBSD phase map (see Figure 9a), the blue regions represent the tungsten matrix, which constitutes a volume fraction of 62.3.% and forms the continuous phase. The light blue regions represent the AlCoCrFeNi_2.1_ reinforcement phase, comprising 15.9% of the material and exhibiting an FCC crystal structure. The red regions represent Al-rich oxides (13.5%), which form around the larger reinforcement particles or small ones dispersed in the matrix. In this case, the addition of Y_2_O_3_ to the model alloy provides nucleation sites for both the reinforcement phases and oxide particles, thereby increasing their volume fraction compared to the Y_2_O_3_-free model alloy (see Figure 6a).

The EBSD orientation map (Figure 9b) reveals a random orientation distribution of the tungsten matrix and the reinforcement phase. The average grain size of the reinforcement phase (d_FCC_ = 0.42 µm ± 0.20) and the tungsten matrix (d_W_ = 0.77 µm ± 0.32) is smaller than that of the W-Al model alloy, as shown in Figure 6b. The EBSD results suggest that the addition of Y_2_O_3_ to the model alloy effectively refines the microstructure and the reinforcement phase. The reinforcement phases are dispersed throughout the tungsten matrix, although some degree of agglomeration is observed, particularly for the larger particles. The smaller reinforcement particles and Al-rich oxide particles are more uniformly distributed. The presence of Y_2_O_3_ nanoparticles serves as a nucleation site for grain refinement, promoting the formation of finer-grained structures. They play a key role in refining the microstructure of the reinforcement phase and encouraging a more uniform distribution of phases.

**Figure 10:**
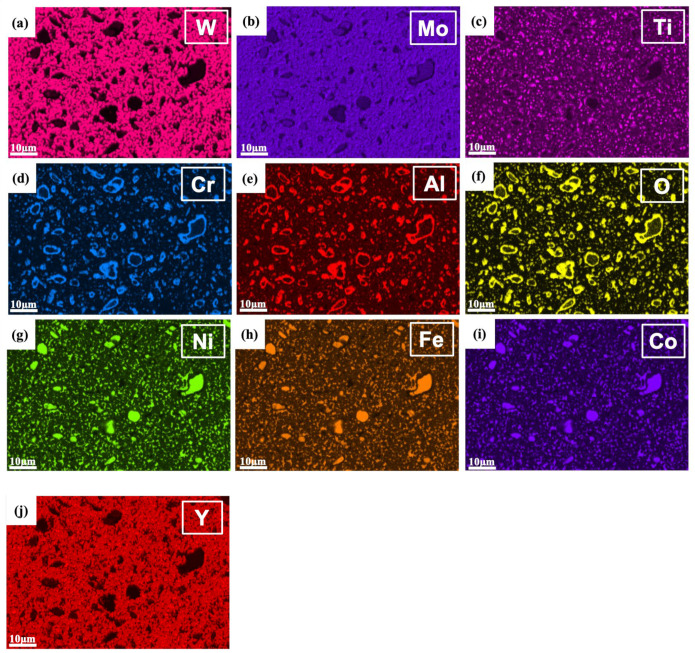
EBSD-EDS mapping of the W-Al-Y model alloy: (**a**) W map, (**b**) Mo map, (**c**) Ti map, (**d**) Cr map, (**e**) Al map, (**f**) O map, (**g**) Ni map, (**h**) Fe map, (**i**) Co map, and (**j**) Y map.

Figure 10 shows the EDS mapping of W-Al-Y model alloys. The AlCoCrFeNi_2.1_ reinforcement phase is readily distinguished by the high concentrations of Ni, Fe, and Co elements. The elevated levels of Al, Cr, and O confirm the presence of the (Al,Cr) oxide phase, which is clearly observed surrounding the larger reinforcement particles. The Ti EDS map also reveals the formation of small Ti-rich oxides, which are homogeneously dispersed within the tungsten matrix. It should be noted that the Y EDS map does not show high concentrations of yttrium (see Figure 10j). This observation suggests that the Y_2_O_3_ particles are likely nanoscale in size, making them challenging to detect or clearly observe using EDS analysis alone.

### 3.4. XRD of Tungsten Model Alloys

**Figure 11:**
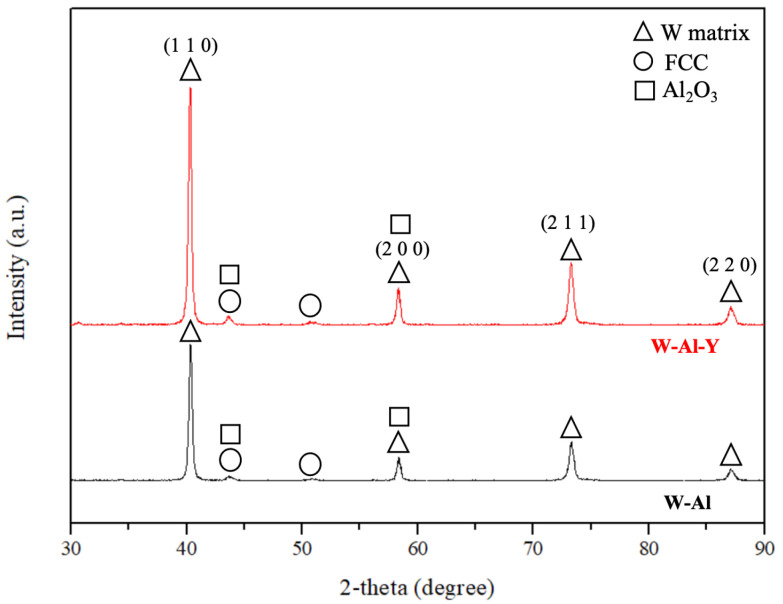
XRD spectra of the tungsten model alloys.

Figure 11 shows the bulk XRD patterns of the tungsten model alloys (W-Al and W-Al-Y), revealing strong peaks corresponding to the tungsten matrix. These peaks are associated with the (110), (200), (211), and (220) planes of the tungsten crystal structure. The FCC reinforcement phase is also observed in the XRD patterns of the tungsten model alloys, as indicated by the weak peaks at 2θ angles of approximately 43° and 51°, corresponding to the (111) and (200) planes of the AlCoCrFeNi_2.1_ alloy, respectively. It should be noted that the Al_2_O_3_ phase was also detected at around 2-theta ~43°, overlapping with the FCC reinforcement phase. On the other hand, the XRD pattern of the W-Al-Y model alloy shows an increase in peak intensity. The phenomenon may be related to grain refinement and improved homogeneity in the alloy, attributed to the dispersion of Y_2_O_3_ particles, which contribute to the stabilization and refinement of the microstructure.

### 3.5. Hardness of Tungsten Model Alloys

**Figure 12:**
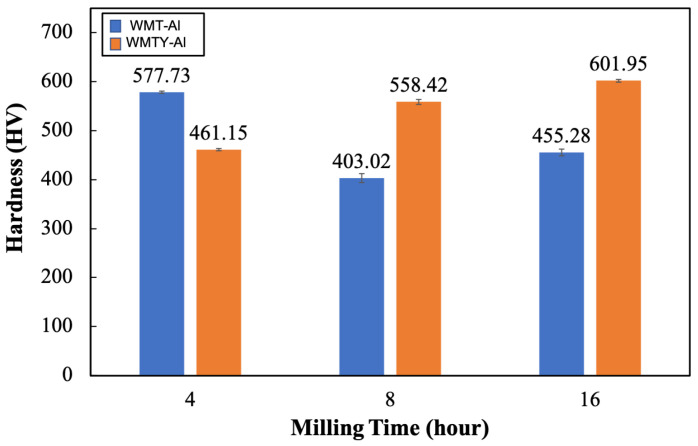
The microhardness of the tungsten model alloys.

Figure 12 shows the Vickers hardness of tungsten model alloys (W-Al and W-Al-Y) at various ball milling times. At 4 h of milling, the W-Al alloy exhibits a higher hardness (577.73 HV) compared to the W-Al-Y alloy (461.15 HV). The higher hardness in W-Al can be attributed to the finer grain structure of the reinforcement phase, which is not agglomerated at this stage. In contrast, the W-Al-Y alloy exhibits lower hardness due to the formation of (Al,Cr) oxides around larger reinforcement phases. These oxide layers act as weak interfaces, reducing the bonding strength between the reinforcement and the matrix. After 8 h of milling, there is a significant drop in the hardness of W-Al (403.02 HV). It suggests that the agglomeration of reinforcement phases with the surrounding (Al,Cr) oxides can impact the hardness of alloys. Meanwhile, W-Al-Y shows an increase in hardness to 558.42 HV in this stage due to a more even distribution of reinforcement phases without excessive clustering. At the final stage of milling, the W-Al alloy has continued growth of large reinforcement phases and the development of a thicker (Al,Cr) oxide layer surrounding them, resulting in a further decrease in hardness to 455.28 HV. However, the hardness of W-Al-Y increases significantly to 601.95 HV, indicating that prolonged milling has led to further refinement of the reinforcement phases and the dispersion of small oxide particles throughout the tungsten matrix. The microstructural refinement and uniform dispersion contribute to the improved hardness of W-Al-Y at this stage.

### 3.6. Compressive Tests of Tungsten Model Alloys

**Figure 13:**
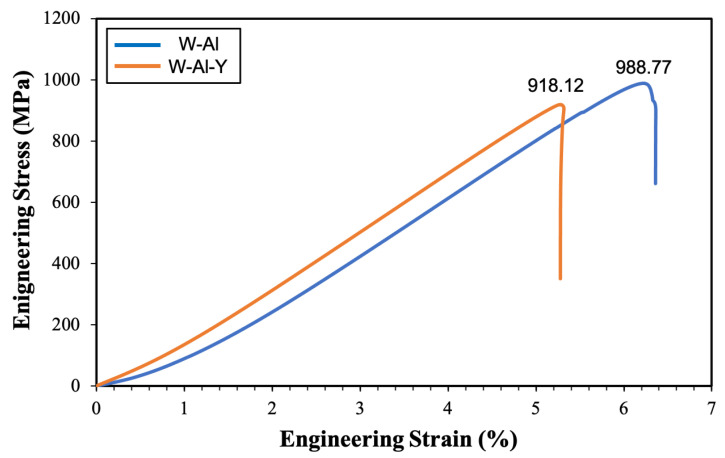
Compressive engineering stress–strain curves of the tungsten model alloys.

Figure 13 shows the compressive stress–strain curves of the W-Al and W-Al-Y model alloys. Both model alloys exhibit a compressive stress of approximately 950 MPa and a compressive strain of about 6%, indicating their brittleness and limited capacity for plastic deformation. In this study, the brittleness of materials can be attributed to the following two points. Firstly, the (Al,Cr) oxide layer surrounding the reinforcement phases significantly affects the mechanical properties. The (Al,Cr) oxide layer may weaken the interfacial bonding of the alloys by introducing brittle regions, which can serve as initiation points for crack formation and propagation under stress. The oxide layer also acts as a brittle barrier around the reinforcement phases, impeding plastic deformation through dislocation movement and consequently reducing ductility. Secondly, the (Al,Cr) oxide layers also have weak interfaces and limited load transfer capabilities. These layers hinder effective load transfer between the reinforcement and the matrix, preventing the reinforcement phases from fully contributing to the alloy’s strengthening. Consequently, the alloy reveals reduced compressive strength and an increased tendency for brittle fracture.

### 3.7. Fracture Analysis

**Figure 14:**
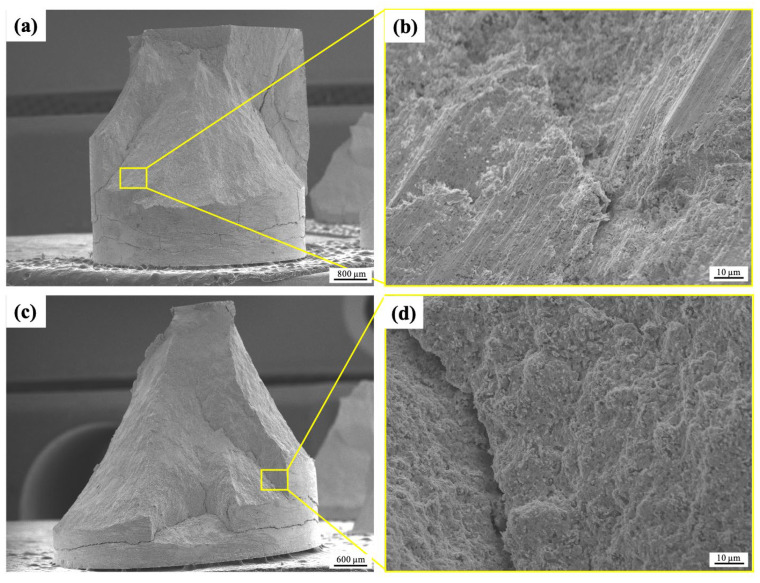
Fracture surfaces of the (**a**,**b**) W-Al and (**c**,**d**) W-Al-Y model alloys after compression tests.

Figure 14 displays the SEM images of the fracture surfaces of W-Al and W-Al-Y alloys following compressive tests. Both W-Al and W-Al-Y show brittle fracture surfaces. In W-Al, the fracture surface demonstrates a textured appearance, indicating a gradual rupture with nonuniform fracture propagation. It may be related to variations in the distribution or interactions of reinforcement phases and (Al,Cr) oxide layers. In this case, the brittle oxide layers may lead to localized crack initiation and propagation in the alloy. On the other hand, the fracture surface of the W-Al-Y alloy appears to have a rough and irregular topography, which may suggest a brittle fracture mechanism with transgranular fracture characteristics. Such uneven textures are typically associated with complex stress distributions during fracture. In this case, the uniform distribution of small reinforcement phases and oxide particles can assist in impeding crack propagation. However, if the interfaces between these small particles and the matrix are weak, they can serve as preferential sites for crack nucleation, contributing to a transgranular fracture pattern.

## 4. Conclusions

This study presents a novel investigation into the role of dispersed Y_2_O_3_ nanoparticles in the microstructural evolution and mechanical properties of tungsten-based alloys reinforced with AlCoCrFeNi_2.1_ phases. The findings highlight the critical influence of Y_2_O_3_ in refining the microstructure and improving phase dispersion, particularly by promoting fine-grained structures in both the tungsten matrix and reinforcement phases. Additionally, this study identifies aluminum depletion and the subsequent formation of (Al,Cr) oxide layers as key factors that hinder the development of the AlNi BCC phase and influence mechanical behavior. A significant distinction is revealed between Y_2_O_3_-containing (W-Al-Y) and Y_2_O_3_-free (W-Al) alloys, with the former demonstrating superior hardness due to enhanced microstructural refinement and phase uniformity, while the latter suffers from reduced hardness caused by agglomeration and oxide thickening. This research further interprets the brittle nature of these alloys, with the (Al,Cr) oxide layers acting as weak interfaces that impair load transfer and limit ductility. This comprehensive understanding of oxide particle effects provides valuable insights for designing advanced tungsten-based composite materials with tailored properties.

## Figures and Tables

**Table 1:** Chemical composition of the model alloys (wt.%).

Materials	W	Mo	Ti	Y_2_O_3_	Al	Co	Cr	Fe	Ni
AlCoCrFeNi_2.1_	-	-	-	-	8.50	18.60	16.40	17.60	38.90
W-Al	81.00	3.00	1.00	-	1.28	2.79	2.46	2.64	5.84
W-Al-Y	79.00	3.00	1.00	2.00	1.28	2.79	2.46	2.64	5.84

## Data Availability

The original contributions presented in this study are included in the article. Further inquiries can be directed to the corresponding author.
